# Breaking barriers in ICD classification with a robust graph neural network for hierarchical coding

**DOI:** 10.1038/s41598-025-10590-1

**Published:** 2025-07-16

**Authors:** Suyang Xi, Jiesen Shi, Jiachen Yan, MingJing Lin, Xinyi Zhou, Yuan Cheng, Hong Ding, Chia Chao Kang

**Affiliations:** 1 School of Artificial Intelligence and Robotics, Xiamen University Malaysia, Sepang, Malaysia; 2 School of Communication, Xiamen University Malaysia, Sepang, Malaysia; 3 School of Life and Health Sciences, Hainan University, Haikou, China

**Keywords:** Outcomes research, Machine learning

## Abstract

The accurate classification of International Classification of Diseases (ICD) codes is a complex and critical multi-label task in clinical documentation, involving the assignment of diagnostic codes to medical discharge summaries. Existing automated methods face challenges due to the sparsity and nuanced nature of medical text, while traditional backpropagation-based models often lack flexibility and robustness. To address these issues, we propose Labeled Graph Generation with Node Representation Grasp (LGG-NRGrasp), an advanced adversarial learning framework that models ICD coding as a labeled graph generation problem. By leveraging a hierarchical structure to refine feature learning, our approach addresses the issue of over-smoothing in deep graph neural networks. A key innovation of LGG-NRGrasp is the integration of adversarial reinforcement learning and domain adaptation techniques, which enhance its ability to generalize across heterogeneous datasets. Extensive evaluations on benchmark datasets indicate that LGG-NRGrasp markedly surpasses leading models, exhibiting enhanced performance and dependability in automated ICD coding.

## Introduction

Clinical documentation spans an extensive range of patient-related data, including admission records, clinical assessments, medical histories, and laboratory findings^1,2^. A critical challenge within this domain is the complex and sometimes contradictory nature of diagnostic coding, as illustrated by codes “783.1” (excessive weight gain) and “783.2” (significant weight loss and underweight). Accurate coding of these conditions plays a vital role in healthcare systems, influencing clinical decision-making, billing, and epidemiological research. However, contemporary automated ICD coding frameworks predominantly employ unified training paradigms for parameter tuning^3–5^, which often exhibit suboptimal performance when faced with nuanced and sparse clinical narratives, particularly those involving rare or less-studied conditions. This limitation arises from a lack of flexibility in modeling the multifaceted interdependencies inherent in medical data, leading to challenges in both scalability and generalizability. To address these challenges, computational approaches capable of modeling structured and interconnected data relationships are essential.

In clinical settings, erroneous or incomplete ICD coding can lead to substantial downstream consequences. For example, in intensive care scenarios captured within the MIMIC-III dataset, a discharge summary that described symptoms of both significant weight loss and malnutrition was occasionally assigned only a general symptom code (e.g., “783.0” – anorexia), missing critical diagnostic codes such as “263.9” (unspecified protein-calorie malnutrition). This omission can result in the underestimation of patient severity and misclassification in Diagnosis-Related Group (DRG) billing systems, which directly impacts hospital reimbursement. Another common instance involves congestive heart failure (CHF), where a patient with “428.0” (congestive heart failure, unspecified) is not properly linked to related comorbidities like “518.81” (acute respiratory failure), causing inadequate risk adjustment in clinical decision-making systems and potential misguidance in treatment prioritization. These examples underscore that even marginal discrepancies in ICD coding can propagate into systemic inefficiencies, affecting both patient outcomes and institutional accountability. Thus, enhancing the fidelity and consistency of ICD code assignment is of paramount importance.

Graph-based methods, particularly Graph Neural Networks (GNNs), offer a promising solution by leveraging their ability to model the complex, interconnected structures inherent in clinical narratives. By encoding relationships among entities, GNNs can potentially address key challenges in ICD coding, such as preserving subtle distinctions between diagnostic categories and accommodating sparse or irregular data. However, despite their potential, GNNs face notable limitations. One critical issue is over-smoothing, where excessive propagation through layers results in the convergence of node representations, erasing crucial distinctions needed for fine-grained classification^6^. Additionally, GNNs are sensitive to structural inconsistencies, including noisy connections and missing data–conditions that are common in medical datasets. Furthermore, in semi-supervised learning scenarios, where labeled data is scarce, GNNs are prone to overfitting, reducing their capacity to generalize effectively. These limitations highlight the need for advanced frameworks capable of enhancing robustness and preserving critical distinctions in complex medical applications.

To address these challenges, recent methods have integrated advanced techniques to enhance GNN performance in complex domains like ICD coding. Regularization strategies, such as DropEdge and node feature augmentation, selectively omit edges or apply dropout to node features, effectively mitigating over-smoothing while preserving distinctive node representations across layers^7,8^. Attention mechanisms dynamically weigh node interactions, emphasizing relevant dependencies within graph structures and improving interpretability^8,9^. Multi-view and multi-scale GNN architectures further enable hierarchical relationship modeling, adapting to varying levels of graph complexity^10,11^. In scenarios with limited or sparse data, reinforcement learning optimizes node representations adaptively, while adversarial training enhances robustness by introducing controlled perturbations to input data^12^, improving resilience to noise and incomplete information^13–15^. Despite these advancements, existing frameworks remain constrained in capturing the multi-dimensional dependencies inherent in clinical data, underscoring the need for a more integrated and flexible solution.

In this paper, we introduce LGG-NRGrasp, a robust and innovative framework for automated ICD coding that redefines the task through a graph-theoretical perspective. The complexity of ICD coding lies in the inherently sparse, interdependent, and highly nuanced nature of clinical data, which often encapsulates intricate relationships among diagnostic codes, hierarchical dependencies, and contextual variability. Traditional models frequently fail to capture these subtleties, leading to issues such as limited accuracy, over-smoothing of features, and poor generalization to noisy or incomplete datasets. To address these challenges, LGG-NRGrasp integrates three pivotal innovations: a Graph-Based Label Generation Mechanism, which structures raw clinical data into relational graphs to uncover intricate dependencies and co-occurrences; a Dynamically Adaptive Architecture, designed to stabilize information flow, mitigate feature over-smoothing, and preserve granularity across sparse and diverse data distributions; and Adversarial Learning Techniques, which align feature representations across domains, enhancing resilience to domain shifts, noise, and missing information. Together, these components enable LGG-NRGrasp to effectively model the relational complexity of medical narratives, ensuring improved stability, accuracy, and adaptability in real-world clinical settings. Empirical evaluations demonstrate its superior performance across varied benchmark datasets, underscoring its potential to set new standards in automated ICD coding. Our main contributions are as follows:

1. *Graph-based label generation framework*: We construct labeled graphs from clinical data, capturing intricate dependencies among diagnostic codes to improve the model’s capacity for accurate ICD classification.
2. *Adaptive model architecture*: We employ residual propagation and feature augmentation to maintain stable information flow and handle sparse, diverse clinical data with precision.
3. *Adversarial training*: We enhance robustness by aligning feature representations, ensuring reliable ICD predictions under noise, missing data, and domain variability.
4. *Theoretical and experimental validation*: We demonstrate the framework’s superiority through rigorous theoretical analysis and comprehensive experiments, showcasing improved performance, stability, and generalization over existing methods.

## Related work

Automated ICD coding has advanced considerably through the integration of deep learning, bridging the gap between unstructured clinical narratives and structured diagnostic codes. Despite these advancements, two major challenges persist: (1) modeling long-range dependencies within lengthy clinical texts, and (2) addressing the hierarchical complexity of ICD codes, which encode taxonomical relationships critical for accurate classification. Early approaches primarily employed Convolutional Neural Networks (CNNs) and attention mechanisms, leveraging their strengths in capturing localized features from clinical text^16–19^. While CNNs effectively extract semantic patterns from shorter text spans, they struggle to capture the long-range dependencies required for extensive clinical narratives. To address this limitation, attention-based models, particularly transformers, emerged as a preferred solution, dynamically focusing on relevant content within complex medical records and demonstrating superior performance over CNN-based methods. However, these sequential frameworks often overlook the hierarchical structure inherent in ICD codes, which is crucial for modeling taxonomical relationships. To overcome this limitation, the Tree-of-Sequences LSTM^20^ was introduced. By explicitly capturing parent-child hierarchies within the ICD taxonomy, this approach enhances both specificity and generalization in ICD classification tasks, marking a significant step forward in addressing the structural complexity of ICD coding.

Graph Representation Learning (GRL) has introduced techniques for embedding graph structures, particularly valuable for ICD coding, where modeling relational dependencies and capturing clinical context are essential. GRL methods, such as translation distance models (e.g., TransE^21^) and semantic matching models (e.g., RESCAL^22^, DistMult^23^, and HoLE^24^), represent relationships in vector spaces or use tensor-based interactions. However, these methods often face limitations in scalability and hierarchical modeling required for ICD taxonomies. Random walk-based methods, like node2vec^25^ and LINE^26^, embed nodes by capturing both structural and contextual information, but lack the hierarchical representation needed for taxonomical relationships. Advanced approaches, such as CompGCN^27^ and TransGCN^28^, extend these frameworks to integrate heterogeneous relationships and support unified graph structures. Moreover, GraphSAGE^29^ provides scalability for large clinical datasets while preserving relational and contextual dependencies. These methods address the challenges inherent in ICD coding, such as capturing hierarchical dependencies and modeling cross-code relationships, making them highly applicable for improving specificity and generalization.

Graph Neural Networks (GNNs) have significantly advanced semi-supervised learning on graph-structured data by capturing intricate relational patterns, surpassing traditional methods such as graph Laplacian regularization^2,20,30^. For ICD coding tasks, GNN methods have progressively addressed key challenges like hierarchical modeling, graph sparsity, and structural noise. The introduction of Graph Convolutional Networks (GCNs)^30^ was a foundational step, leveraging iterative neighborhood aggregation to model complex graph relationships. However, GCNs’ sensitivity to sparse and noisy graph structures hindered their effectiveness in ICD coding scenarios. Subsequent methods like GraphSAGE^3^ incorporated neighborhood sampling, which not only mitigated overfitting but also provided robustness against sparsity by aggregating features from diverse node subsets. To address scalability while maintaining structural coherence, approaches like Cluster-GCN^5^ utilized graph partitioning for efficient training on large-scale hierarchical graphs. Meanwhile, decoupled architectures^6^ improved noise resilience by separating feature propagation from non-linear transformations, providing more stable and interpretable predictions.

## Methodology

**Fig. 1:**
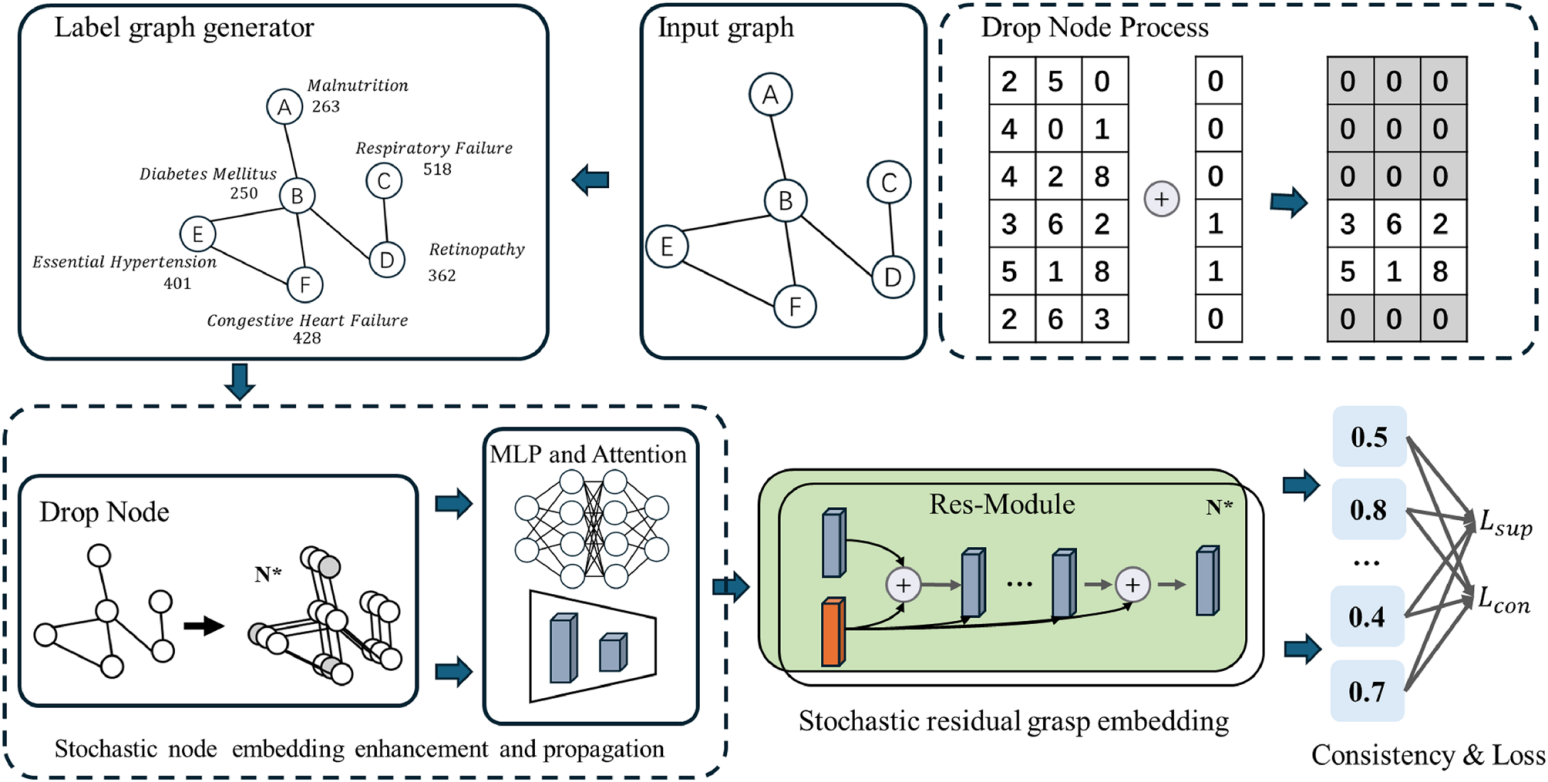
End-to-end architecture of LGG-NRGrasp for robust ICD Code prediction. The LGG-NRGrasp framework combines a reinforcement learning-based *Label Graph Generator* with the original clinical input graph to construct a dynamic label dependency graph tailored for ICD coding. This is followed by *Stochastic Node Embedding Enhancement and Propagation*, which introduces DropNode perturbations and aggregates multi-hop features via a mixed-order adjacency matrix to enhance robustness. Subsequently, the *Stochastic Residual Grasp Embedding* module preserves discriminative features using residual propagation across depth-varying layers. Finally, multiple augmented views are supervised through a joint optimization of supervised loss and consistency regularization, resulting in stable and generalizable ICD code predictions across both labeled and unlabeled nodes.

We introduce LGG-NRGrasp, an innovative method for automated ICD coding that combines reinforcement learning, graph neural networks, and adversarial training inside a comprehensive and adaptable framework. As shown in Fig. 1, this algorithm structure utilizes these sophisticated strategies to guarantee the model attains enhanced coding accuracy while preserving robustness against structural anomalies and domain-specific obstacles. We provide a thorough theoretical study to confirm these contributions, emphasizing the framework’s convergence guarantees, generalization ability, and resilience.

### Label graph generator

*Motivation:* In the context of ICD coding, effectively capturing intricate relational dependencies among clinical labels is critical. Given that the graph structure is inherently sparse and evolves over time, we aim to use reinforcement learning to dynamically build the label graph while prioritizing long-term prediction accuracy. The probabilistic decision-making process helps adapt to these complexities, ensuring robustness to various graph configurations.

*Specification:*In graph neural networks (GNNs), effective label generation is a critical component, particularly in applications like ICD coding, where capturing intricate relational dependencies among clinical labels is essential. Within our LGG-NRGrasp framework, we address this challenge by leveraging a parameterized policy $\pi _\theta$ that enables probabilistic decision-making over state-action pairs, adapting dynamically to the complexities of graph-structured clinical data. Specifically, in our approach, $s$ represents the current graph state, which includes the partially constructed label graph $G_t$ at step $t$, including the node features and existing edge connections. The action $a$ denotes a discrete decision, such as adding or removing an edge between two nodes, i.e., $a_t = (v_i, v_j, \text {add/remove})$. The policy is parameterized as $\pi _\theta (a|s, X) = \sigma (W_\theta s + b_\theta )$, where $W_\theta$ and $b_\theta$ are trainable parameters, $\sigma$ is a sigmoid activation function, and $X$ represents the contextual feature set encoding the current node pair and graph topology. Here, $\sigma$ maps linear outputs into a probabilistic space, essential for guiding the policy as it constructs the label graph edge-by-edge within the ICD coding task.

By formulating the policy gradient based on the expected cumulative return over trajectories $\tau = \{(s_0, a_0), \dots , (s_T, a_T)\}$, defined as $R(\tau ) = \sum _{t=0}^T \gamma ^t R(s_t, a_t)$, with $\gamma$ as a discount factor, LGG-NRGrasp effectively prioritizes long-term accuracy in label prediction. The reward function $R(s_t, a_t)$ is designed to reflect the structural quality of the generated graph, including sparsity regularization and downstream label prediction performance. The probability density $p_\theta (\tau )$ captures the distribution of trajectories shaped by the policy, allowing us to optimize the expected return $J(\theta )$ relative to policy parameters $\theta$, which is mathematically expressed as:

1 $$\begin{aligned} \nabla _\theta J(\theta ) = \nabla _\theta \mathbb {E}_{\tau \sim p_\theta (\tau )}[R(\tau )] = \int _\tau \nabla _\theta p_\theta (\tau ) R(\tau ) d\tau = \int _\tau p_\theta (\tau ) \nabla _\theta \log p_\theta (\tau ) R(\tau ) d\tau = \mathbb {E}_{\tau \sim p_\theta (\tau )}\left[ \nabla _\theta \log p_\theta (\tau ) R(\tau ) \right] \end{aligned}$$

The decomposition of $\log p_\theta (\tau )$ as $\sum _{t=0}^T \log \pi _\theta (a_t | s_t) + \log P(s_{t+1}|s_t, a_t)$ reveals that $P(s_{t+1}|s_t, a_t)$, which represents the deterministic graph transition given an action (i.e., updating $G_t$ with the new edge decision), is independent of the parameters $\theta$, resulting in its gradient vanishing. Thus, the gradient of $J(\theta )$ is governed solely by the policy $\pi _\theta$. To compute this gradient efficiently, we employ the REINFORCE algorithm, a foundational policy gradient method that enables stochastic optimization of $J(\theta )$. This algorithm iteratively adjusts $\theta$ to maximize expected return, with the gradient represented as:

2 $$\begin{aligned} \nabla _\theta J(\theta ) = \mathbb {E}_{\tau \sim p_\theta (\tau )}\left[ \sum _{t=0}^{T} \nabla _\theta \log \pi _\theta (a_t|s_t) \left( \sum _{t'=t}^{T} \gamma ^{t'-t} R(s_{t'}, a_{t'}) \right) \right] \end{aligned}$$

The discount factor $\gamma$ modulates the temporal significance of rewards, ensuring future returns are appropriately weighted within the trajectory. This formulation captures the influence of action $a_t$ taken at time step *t* on the trajectory’s cumulative return, indicating how policy refinements impact performance. The trajectory $\tau = (s_0, a_0, s_1, a_1, \ldots , s_T, a_T)$ evolves by progressively modifying the graph structure based on actions sampled from the policy, with $\gamma$ balancing long-term rewards. To practically estimate this gradient, we introduce an unbiased estimator:

3 $$\begin{aligned} \hat{g} = \sum _{t=0}^T \nabla _\theta \log \pi _\theta (a_t|s_t) \sum _{t'=t}^T \gamma ^{t'-t} R(s_{t'}, a_{t'}) \end{aligned}$$

where $\hat{g}$ directly leverages the state-action pairs across sampled graph trajectories, evaluating the contribution of each edge decision to the total return at every time step. Taking the expectation over these trajectories yields $\mathbb {E}_{\tau \sim p_\theta (\tau )}[\hat{g}] = \nabla _\theta J(\theta )$, confirming that $\hat{g}$ serves as an unbiased estimator of the policy gradient. This ensures robust convergence in optimizing $\theta$, with the REINFORCE algorithm providing an efficient mechanism for enhancing the quality of generated label graphs and overall performance of the GNN in ICD label prediction tasks.

### Stochastic node embedding enhancement and propagation

*Motivation:* To address the challenge of over-smoothing in deep GNNs, we introduce DropNode, which operates at the feature level instead of the graph structure level. By applying noise at the node feature level, this technique ensures that the model avoids over-reliance on specific nodes and improves the robustness of learned embeddings, especially in sparse graph structures like those found in clinical data.

*Specification:* The propagation process in our proposed framework is divided into two key stages. The first stage involves introducing noise into the original feature matrix *X* through a random dropout mechanism, resulting in a perturbed feature matrix $\tilde{X}$. This perturbation selectively masks elements of *X* to dynamically alter node features. Subsequently, $\tilde{X}$ is subjected to feature propagation, producing augmented features denoted as $\bar{X}$. The stochastic nature of this perturbation process allows each node’s feature distribution to be adaptively enriched by aggregating information from its neighbors, following the homophily principle, which suggests that adjacent nodes in a graph are more likely to share similar attributes and labels^31^. Consequently, any missing or incomplete data for a node can be inferred from its neighborhood, thereby improving the overall quality of node representations.

Several strategies can be adopted to perturb *X* during training. A widely used approach is dropout^32^, which operates by randomly setting individual elements of *X* to zero, thereby forming the perturbed matrix $\tilde{X}_{ij} = \frac{\epsilon _{ij}}{1-\delta } X_{ij}$, where $\epsilon _{ij}$ is sampled from a Bernoulli distribution $Bernoulli(1-\delta )$. This method injects noise independently of the graph structure, effectively regularizing the model. However, to better account for the graph’s intrinsic structural dependencies, we propose an alternative technique called *DropNode*. Rather than masking single feature entries, DropNode applies a mask to entire node feature vectors, allowing nodes to aggregate information from a subset of multi-hop neighbors. While the concept of node-level dropout has appeared in prior works such as DropEdge^7^, our DropNode method is uniquely designed to operate in the node feature masking space rather than altering the graph topology. Specifically, instead of modifying the edge structure, we stochastically drop entire node embeddings during feature perturbation. This enables a stronger form of semantic-level augmentation, encourages feature diversity, and prevents feature co-adaptation during propagation. Empirical results demonstrate that DropNode produces more diverse data augmentations and outperforms conventional dropout in terms of model accuracy. During training, the perturbed matrix $\tilde{X}$ is rescaled to preserve its expected equivalence to the original feature matrix *X*, while *X* remains unchanged during inference to ensure stable predictions.

In the second stage of propagation, we implement a mixed-order feature propagation mechanism, represented as $\bar{X} = \bar{A}\tilde{X}$. The mixed adjacency matrix $\bar{A}$ is constructed by integrating multiple powers of the normalized adjacency matrix $\hat{A}$, specifically as $\bar{A} = \sum _{k=0}^K \frac{1}{K+1} \hat{A}^k$. By integrating graph structure information from local to global scales, this formulation effectively mitigates the over-smoothing effects that typically arise in deep GNNs, preserving meaningful distinctions among node representations^10,33^. To alleviate the computational overhead of directly calculating $\bar{A}$, an iterative approximation technique is used to efficiently compute the matrix product $\bar{A}\tilde{X}$. Furthermore, *DropNode* can be viewed as an operation that omits certain rows from $\bar{A}$, unlike DropEdge^7^, which perturbs the structural adjacency, our DropNode modifies the feature input pipeline by selectively suppressing node-level signals. This allows our method to regularize the network without disrupting topological consistency, making it more suitable for tasks where structural semantics should remain intact, such as ICD code dependency modeling. Perturbation techniques like DropNode and DropEdge can be further refined by adjusting the adjacency matrix $\hat{A}$ and applying mixed-order propagation, serving as a substitute for the initial propagation matrix.

Upon completing random propagation across $S$ instances (where $s$ indexes each individual instance), we obtain $S$ augmented feature matrices, $\{\bar{\textsf{X}}^{(s)} \mid 1 \le s \le S\}$. Each augmented matrix undergoes a dual transformation via concurrent processing through an attention mechanism and a multi-layer perceptron (MLP)^34^. This enhances node representations by leveraging both the local structural context and higher-order feature abstractions. The prediction probabilities for node labels are then computed as follows:

4 $$\begin{aligned} \tilde{\textsf{Z}}^{(s)}&= f_{\text {att-mlp}}\left( \bar{\textsf{X}}^{(s)}, \Phi \right) \end{aligned}$$

where $\tilde{\textsf{Z}}^{(s)} \in [0, 1]^{n \times C}$ denotes the prediction probabilities at the node level, with $n$ representing the number of nodes in the graph and $C$ the number of classes or labels for each node, and $\Phi$ represents the trainable parameters of the model. Unlike standard GNNs, which disentangle the propagation and transformation processes, our framework concurrently integrates attention mechanisms and MLP transformations. The attention module dynamically captures inter-node dependencies and emphasizes critical structural relationships, while the MLP refines node embeddings through non-linear transformations, capturing complex feature hierarchies. This integrated approach balances context-sensitive propagation and effective generalization, reducing over-smoothing effects in deep GNNs and producing robust node representations that generalize well across diverse graph structures.

### Stochastic residual grasp embedding

*Motivation:* In deep GNNs, over-smoothing and loss of information due to propagation across many layers can degrade performance. The introduction of Stochastic Residual Grasp Embedding (SRGE) ensures that residual features are preserved and integrated progressively, balancing between initial node features and propagated information. This prevents excessive dilution of node representations, which is critical for tasks where fine-grained feature extraction is needed.

*Specification:* We present a novel graph neural network (GNN) architecture underpinned by the Stochastic Residual Grasp Embedding approach, aimed at effectively mitigating the common issue of over-smoothing in deep GNNs. This architecture integrates three core mechanisms: Adjacency Matrix Normalization (AMN), MASK-based Multiple Residual Propagation (MMRP), and the Res module within Deeper Map Representation Modeling (DMRM), each contributing to robust embedding learning and improved model expressiveness.

In our framework, the Adjacency Matrix Normalization (AMN) plays a crucial role by stabilizing the propagation of features through a novel normalization approach. This is achieved by appropriately scaling the adjacency matrix to mitigate potential over-smoothing effects that arise from uncontrolled propagation dynamics in deeper networks. Specifically, the normalized adjacency matrix is given by:

5 $$\begin{aligned} \hat{A}&= (D + I)^{-1/2}(A + I)(D + I)^{-1/2} \end{aligned}$$

where *A* denotes the adjacency matrix, *D* is the degree matrix, and *I* represents the identity matrix. This normalization adjusts the influence of neighboring nodes based on their degrees, maintaining a balanced spectral distribution. The spectral properties of $\hat{A}$ are crucial as they prevent the exponential growth of propagated features, ensuring numerical stability. To understand this normalization’s effectiveness, consider the eigenvalues of $\hat{A}$. For any eigenvalue $\lambda$ associated with its eigenvector *v*, we have: $\lambda v = (D + I)^{-1/2}(A + I)(D + I)^{-1/2}v$, where $\lambda$ is an eigenvalue of $\hat{A}$. Rewriting and letting $u = (D + I)^{-1/2}v$ (a transformed eigenvector of $\hat{A}$), we transform the relationship into: $\lambda (D + I) u = (A + I) u$. From here, it follows that:

6 $$\begin{aligned} \lambda \sum _j (d_j + 1) u_j^2 = \sum _{i,j} (A_{ij} + \delta _{ij}) u_i u_j \end{aligned}$$

where $d_j$ denotes the degree of node *j* and $\delta _{ij}$ is the Kronecker delta. Bounding the right-hand side through careful summation of contributions from neighboring nodes, we obtain the inequality: $\left| \sum _{i,j} (A_{ij} + \delta _{ij}) u_i u_j \right| \le \sum _j (d_j + 1) u_j^2$, thereby confirming that $|\lambda | \le 1$. This bound ensures all eigenvalues of $\hat{A}$ fall within the interval $[-1, 1]$, preserving the necessary stability for effective graph propagation and preventing over-smoothing.

To combat the over-smoothing phenomenon, MMRP employs a residual propagation mechanism that retains and progressively updates initial node features while executing multi-step aggregation. The MMRP operation is defined as $H^{(l+1)} = (1 - \alpha ) \hat{A} H^{(l)} + \alpha H^{(0)}$, where $\alpha \in (0, 1)$ is a balancing hyperparameter that regulates the mix between the initial features $H^{(0)}$ and the propagated features at layer *l*. The MMRP process ensures convergence to a unique fixed point $H^{(\infty )} = \alpha (I - (1 - \alpha ) \hat{A})^{-1} H^{(0)}$, thereby achieving robust and stable propagation. Moreover, the convergence to this fixed point happens at an exponential rate, characterized by:

7 $$\begin{aligned} \Vert H^{(l)} - H^{(\infty )} \Vert&\le (1 - \alpha )^l \Vert H^{(0)} - H^{(\infty )} \Vert \end{aligned}$$

indicating rapid convergence influenced by the parameter $\alpha$. This allows for deep feature integration without excessive dilution, effectively mitigating over-smoothing. DMRM further enhances the expressive capacity of the model by iteratively refining node features, incorporating varying weights for each layer to adapt to the complexity of the graph structure. This operation is captured as:

8 $$\begin{aligned} H^{(l)} = (1 - \alpha _l) \hat{A} H^{(l-1)} W_1 + \alpha _l H^{(0)} W_l \end{aligned}$$

where $\alpha _l = \log \left( \frac{l}{l + 1} \right) + \epsilon$ is a layer-specific coefficient that dynamically adjusts transformation depth, and $\epsilon$ is a small hyperparameter for fine-tuning. Our adaptive weighting mechanism allows DMRM to equilibrate the influence of transmitted features while maintaining crucial starting embeddings. Utilizing the universal approximation theorem, DMRM approximates any continuous function on a compact domain, adeptly simulating intricate brain changes and maintaining graph structure via customized propagation and weighting.

### Consistency regularized training

*Motivation:* Semi-supervised learning in graph settings often suffers from high variance due to limited labeled data. Consistency regularization is introduced to mitigate this, ensuring that model predictions are stable across different augmentations of the same data. By enforcing this consistency, we can achieve better generalization, especially when labeled data is scarce.

*Specification:* In semi-supervised graph learning, the objective is to smooth label propagation by integrating supervised loss and regularization. Our approach, LGG-NRGrasp, uses random data augmentation and consistency regularization to enhance generalization with limited labeled data. Given *S* augmentations generated during propagation, the supervised loss is defined as $L_{\text {sup}} = -\frac{1}{S} \sum _{s=1}^S \sum _{i=0}^{m-1} Y_i^\top \log Z_i^{(s)}$, where $Y_i$ represents the one-hot encoded true label vector for node *i*, and $Z_i^{(s)}$ is the predicted label probability distribution for the *s*-th augmentation. For consistency regularization, we align predictions across augmentations to minimize variance: $L_{\text {con}} = \frac{1}{S} \sum _{s=1}^S \sum _{i=0}^{n-1} \Vert \bar{Z}_i - Z_i^{(s)} \Vert ^2$, where $\bar{Z}_i = \frac{1}{S} \sum _{s=1}^S Z_i^{(s)}$ is the averaged distribution across augmentations. To encourage confident predictions and reduce uncertainty, we apply a sharpening operation to the predicted label distributions. Let $Z_{ij}$ represent the probability of the *i*-th node belonging to the *j*-th class before sharpening. We introduce a temperature parameter *T* to control the distribution’s sharpness. The sharpening is formulated as follows:

9 $$\begin{aligned} Z_{ij}' = \frac{Z_{ij}^{1/T}}{\sum _{c=0}^{C-1} Z_{ic}^{1/T}} \end{aligned}$$

where *T* controls the sharpness. The overall objective function is designed to balance supervised learning and consistency regularization, formulated as $L = L_{\text {sup}} + \lambda L_{\text {con}}$. Here, $\lambda$ acts as a trade-off parameter, regulating the contribution of each term to ensure smooth label propagation and robust generalization across both labeled and unlabeled nodes.

### Adversarial domain adaptation

*Motivation:* In clinical graph learning, source and target domains often exhibit significant structural and semantic discrepancies due to variations in patient populations, hospital coding practices, and data sparsity. Traditional domain adaptation techniques struggle to align these complex distributions, especially when label graphs carry hierarchical or dependency constraints. To address this, we employ adversarial domain adaptation based on the Wasserstein distance, which offers a geometry-aware metric to match distributions with minimal structural distortion. This enables our model to generalize across domains while preserving critical relational information encoded in the label graph.

*Specification:* In our framework, adversarial domain adaptation bridges the gap between source and target domains, which often vary considerably across clinical settings. The source domain refers to training data from a specific dataset or institution, while the target domain represents data from new clinical environments. To enhance generalization across these domains, we use an adversarial approach based on optimal transport, employing the Wasserstein distance to align feature distributions and mitigate domain shift, a common challenge in medical applications. Given two probability measures, $\mu$ for the source domain and $\nu$ for the target domain over a metric space (*X*, *d*), where *X* represents the feature space and *d* denotes the distance metric, the Wasserstein distance of order *p* provides a structured way to quantify the distributional differences between these domains. Formally, the Wasserstein distance is defined as:

10 $$\begin{aligned} W_p(\mu , \nu )&= \left( \inf _{\gamma \in \Pi (\mu , \nu )} \int _{X \times X} d(x, y)^p \, d\gamma (x, y) \right) ^{1/p} \end{aligned}$$

where $\Pi (\mu , \nu )$ denotes the set of all joint distributions with marginals $\mu$ and $\nu$. In our context, this distance serves as a criterion for minimizing domain discrepancy. For $p = 1$ and when $X$ is compact, the Wasserstein distance can be expressed in its dual form, which allows for a practical implementation using an adversarial training approach. The dual representation is:

11 $$\begin{aligned} W_1(\mu , \nu )&= \sup _{f \in \text {Lip}_1(X)} \left| \int _X f \, d\mu - \int _X f \, d\nu \right| \end{aligned}$$

where $\text {Lip}_1(X)$ is the space of 1-Lipschitz continuous functions over $X$. This duality establishes a foundation for an adversarial domain adaptation mechanism, in which we train a generator $G$ and a critic $f$ in a minimax game to reduce the Wasserstein distance between the learned feature distributions $p_s$ (source) and $p_t$ (target). The adversarial objective is formulated as:

12 $$\begin{aligned} \min _G \max _{f \in \text {Lip}_1(X)} \mathbb {E}_{x \sim p_s} [f(x)] - \mathbb {E}_{x \sim p_t} [f(G(x))] \end{aligned}$$

By integrating this domain adaptation strategy with our previously described methods for feature propagation and stochastic node embedding, we create a robust mechanism that not only enhances local feature alignment but also effectively reduces global domain discrepancies. The adversarial framework complements our propagation techniques by continuously refining the embeddings to account for domain shifts, ensuring that the learned representations generalize well across different domains.

The stability and convergence of this adversarial training process are ensured under the conditions of Lipschitz continuity for both $G$ and $f$, along with bounded gradient norms. The optimization dynamics converge to a saddle point of the Wasserstein objective, which is guaranteed by leveraging principles from two-player zero-sum games and stochastic gradient descent-ascent techniques. The convergence proof involves verifying that the gradient updates serve as unbiased estimators of the true gradients and applying the Robbins-Siegmund theorem for almost sure convergence. Letting $L(G, f)$ represent the objective for minimizing the Wasserstein distance, the expected gradient update for the generator is given by:

13 $$\begin{aligned} \mathbb {E} \left[ \nabla _G L(G_t, f_t) \bigg | {F}_t \right]&= \nabla _G L(G_t, f^*(G_t)) \end{aligned}$$

where $f^*(G)$ denotes the optimal critic for a fixed generator $G$, and ${F}_t$ represents the filtration up to iteration $t$. The application of stochastic approximation techniques ensures that the training converges to a local Nash equilibrium. Additionally, the Wasserstein distance’s stability with respect to changes in the generator parameters is crucial. For a generator $G_\theta$ parameterized by $\theta$, the Lipschitz continuity of the distance $W_1(p_s, G_\theta (p_t))$ ensures smooth updates during training:

14 $$\begin{aligned} |W_1(p_s, G_{\theta _1}(p_t)) - W_1(p_s, G_{\theta _2}(p_t))|&\le W_1(G_{\theta _1}(p_t), G_{\theta _2}(p_t)) \nonumber \\ &\le K \mathbb {E}_{x \sim p_t} [\Vert G_{\theta _1}(x) - G_{\theta _2}(x)\Vert ] \nonumber \\ &\le K L \Vert \theta _1 - \theta _2\Vert \end{aligned}$$

where $K$ is the Lipschitz constant for the distance metric over $X$, and $L$ is the Lipschitz constant for the generator $G_\theta$. This ensures that the training process remains stable and that the updates in parameter space lead to a steady convergence. This adversarial domain adaptation framework, when integrated with our propagation and embedding strategies, provides a comprehensive solution for effectively aligning source and target distributions, enhancing the robustness of the learned representations across different domains.

## Theoretical analysis

### Regularization effects of random propagation

In the LGG-NRGrasp framework, random propagation and consistency regularization enhance model robustness and classification accuracy. Our analysis shows that DropNode considerably outperforms traditional dropout by better leveraging graph topology and capturing higher-order relationships. DropNode’s structured perturbation promotes diverse information propagation across multi-hop neighborhoods, resulting in richer feature representations and improved generalization. We analyze these effects in the context of a binary classification task, where the output of the MLP is simplified to a single layer representation, given by $\tilde{\textsf{Z}} = \sigma (\tilde{\textsf{A}} \textsf{X} \textsf{W})$. Here, $\textsf{W}$ denotes the learnable weight matrix, $\tilde{\textsf{A}}$ is the perturbed adjacency matrix capturing the propagated structural information, and $\textsf{X}$ represents the feature matrix. For the *i*th node, the conditional output distribution under perturbation is expressed as $\tilde{z}_i = \sigma _i(1 - z_i)^{1 - y_i}$, where $z_i$ is the predicted probability and $y_i \in \{0, 1\}$ denotes the ground truth label.

The consistency regularization loss can be defined for the scenario where we generate $S = 2$ augmentations. This regularization term penalizes the divergence between the outputs under different augmentations, given by:

15 $$\begin{aligned} L_{\text {con}} = \frac{1}{2} \sum _{i=0}^{n-1} \left( \tilde{z}_i^{(1)} - \tilde{z}_i^{(2)} \right) ^2 \end{aligned}$$

where $\tilde{z}_i^{(1)}$ and $\tilde{z}_i^{(2)}$ represent the model outputs on node *i* for the two augmentations, respectively. By minimizing this consistency loss, we effectively approximate a regularization term of the form: $\mathbb {E}[L_{\text {con}}] \approx R^c(\textsf{W}) = \sum _{i=0}^{n-1} z_i^2 (1 - z_i)^2 \text {Var}(\tilde{\textsf{A}} \textsf{X} \textsf{W})$, where $\text {Var}(\tilde{\textsf{A}} \textsf{X} \textsf{W})$ represents the variance of the propagated feature representations under random perturbations. This variance captures the inherent instability in the feature space due to perturbations, serving as a measure of uncertainty. To derive the form of $\text {Var}(\tilde{\textsf{A}} \textsf{X} \textsf{W})$, we consider the DropNode perturbation mechanism, where random node dropout is applied to $\textsf{X}$. The variance of the perturbed features can be expressed as:

16 $$\begin{aligned} \text {Var}(\tilde{\textsf{A}} \textsf{X} \textsf{W}) = \frac{\delta }{1-\delta } \sum _{j=0}^{J} (\textsf{X}_j \textsf{W})^2 (\tilde{A}_{ij})^2 \end{aligned}$$

with $\delta$ representing the drop rate of the nodes, effectively scaling the influence of node perturbations. Consequently, the corresponding regularization term for DropNode perturbation becomes:

17 $$\begin{aligned} R_{\text {DN}}(\textsf{W}) = \frac{\delta }{1-\delta } \sum _{i=0}^{n-1} \sum _{j=0}^{J} (\textsf{X}_j \textsf{W})^2 z_i^2 (1 - z_i)^2 (\tilde{A}_{ij})^2 \end{aligned}$$

The term $z_i^2 (1 - z_i)^2$ quantifies classification uncertainty, attaining its peak when $z_i = 0.5$, indicating maximum uncertainty, and approaching zero when $z_i = 0$ or 1, signifying high confidence. The multi-hop neighborhood influence is encapsulated by $(\tilde{A}_{ij})^2$, emphasizing the importance of graph topology in regularization. Similarly, considering the dropout method, where features in $\textsf{X}$ are randomly set to zero, the corresponding variance term adjusts to: $\text {Var}(\tilde{\textsf{A}} \textsf{X} \textsf{W}) = \frac{\delta }{1-\delta } \sum _{j=0}^{J} (\textsf{X}_j \textsf{W})^2 (\tilde{A}_{ij})^2$, resulting in a modified regularization expression:

18 $$\begin{aligned} R_{\text {DN}}(\textsf{W}) = \frac{\delta }{1-\delta } \sum _{i=0}^{n-1} \sum _{j=0}^{J} (\textsf{X}_j \textsf{W})^2 z_i^2 (1 - z_i)^2 (\tilde{A}_{ij})^2 \end{aligned}$$

While similar to the DropNode regularization, dropout introduces a distinct amplification of the variance, which corresponds to a different weighting scheme for the classification uncertainty. The dropout regularization effectively acts as an adaptive $L_2$ penalty on $\textsf{W}$, with the strength of the regularization determined by the structure of the graph and the uncertainty in the label space. Moreover, random propagation influences the supervised classification loss. The perturbed supervised loss, accounting for the stochastic propagation, can be formulated as:

19 $$\begin{aligned} L_{\text {sup}} = \sum _{i=0}^{m-1} -y_i \log (\tilde{z}_i) - (1-y_i) \log (1-\tilde{z}_i) \end{aligned}$$

where $\tilde{z}_i$ represents the predicted probability for node *i* after applying perturbation. The original classification loss, without perturbation, is given by $L_{\text {orig}} = \sum _{i=0}^{m-1} -y_i \log (z_i) - (1-y_i) \log (1 - z_i)$, where $z_i = \sigma (\textsf{A} \textsf{X} \textsf{W})$ denotes the output based on the unperturbed features. The impact of random propagation on the supervised loss introduces an implicit regularization effect, equivalent to optimizing the original loss with an additional term: $\mathbb {E}[L_{\text {sup}}] \approx L_{\text {orig}} + R^c(\textsf{W})$, where $R^c(\textsf{W})$ aligns with the quadratic approximation of the consistency regularization term, given by $R^c(\textsf{W}) = \frac{1}{2} \sum _{i=0}^{n-1} z_i (1 - z_i) \text {Var}(\tilde{\textsf{A}} \textsf{X} \textsf{W})$. This regularization effect promotes consistency across predictions by reducing the variance in classification outputs due to perturbations, thus mitigating overfitting and enhancing the generalization capability of the model.

The theoretical insights derived from these analyses reveal that combining random propagation with consistency regularization introduces a robust form of regularization that leverages the graph structure and uncertainty information. The incorporation of multi-hop neighborhood interactions via the variance term $\text {Var}(\tilde{\textsf{A}} \textsf{X} \textsf{W})$ captures the essential graph topology, while the adaptive weighting of classification uncertainty ensures that the model remains resilient to perturbations across various levels of node confidence.

### Generalization performance

Building on our approach to Adversarial Domain Adaptation, we analyze the generalization capabilities of the LGG-NRGrasp model by utilizing principles of algorithmic stability. By aligning the source and target domains through domain adaptation, our goal is to ensure that the model generalizes well when deployed in new environments. To derive generalization bounds for our model, we utilize the principles of algorithmic stability. An algorithm $A$ is defined as $\beta$-uniformly stable if for any datasets $S$ and $S'$ that differ by at most one element, and for any input $x$, the deviation in the algorithm’s output remains bounded:

20 $$\begin{aligned} |A(S)(x) - A(S')(x)| \le \beta \end{aligned}$$

where *A*(*S*)(*x*) and $A(S')(x)$ denote the model’s predictions for input *x* when trained on the source dataset *S* and the target dataset $S'$, respectively. Based on this stability definition, the generalization bound can be formalized. For an algorithm $A$ that is $\beta$-uniformly stable and trained on a dataset $S$ of size $m$, the generalization error between the true loss $L$ and the empirical loss $\hat{L}$ is bounded with high probability as

21 $$\begin{aligned} \mathbb {E}[L(A(S)) - \hat{L}(A(S))] \le \beta + \sqrt{\frac{2 \log (2/\delta )}{m}} \end{aligned}$$

where this bound holds with probability at least $1 - \delta$. The proof utilizes McDiarmid’s inequality alongside the properties of $\beta$-uniform stability. Specifically, let $Z_i = L(A(S^{(i)})) - L(A(S))$, where $S^{(i)}$ differs from $S$ by the $i$-th element. Since $|Z_i| \le \beta$, McDiarmid’s inequality implies:

22 $$\begin{aligned} P\left( \left| L(A(S)) - \hat{L}(A(S))\right| > t\right) \le 2 \exp \left( -\frac{2 t^2}{m \beta ^2} \right) \end{aligned}$$

where setting the probability to $\delta$ and solving for $t$ provides the desired bound. We ascertain the stability qualities of the LGG-NRGrasp model to use this theorem. The LGG-NRGrasp algorithm exhibits $\beta$-uniform stability with $\beta = O\left( \frac{1}{m}\right)$, where *m* denotes the training set size. This stability characteristic implies that the model’s output varies minimally with the alteration of a single training example. The analysis is built upon three key components: evaluating the stability of the graph neural network component, bounding the variations in policy gradient updates, and leveraging the Lipschitz continuity of the Wasserstein distance. The primary insight is that the effect of any individual sample diminishes as the training set size increases, resulting in a bound of $O\left( \frac{1}{m}\right)$. By integrating this stability bound into the generalization bound theorem, the generalization error of LGG-NRGrasp is constrained as

23 $$\begin{aligned} \mathbb {E}[L(A(S)) - \hat{L}(A(S))] \le O\left( \frac{1}{m}\right) + \sqrt{\frac{2 \log (2/\delta )}{m}} \end{aligned}$$

providing a theoretical assurance regarding the generalization capabilities of the model.

### Robustness guarantees

Establishing robustness guarantees for adversarially trained models is crucial for understanding their stability against small input perturbations. These guarantees are grounded in the concept of Lipschitz continuity, which provides a measure of how the output of a function is bounded under small changes to its input. In the context of our model, the robustness properties are linked to the behavior of $f_\theta$, the learned function under adversarial training. For a given model $f_\theta$, trained within an adversarial framework and subjected to a perturbation bound $\epsilon$, the relationship between the input perturbations and output variations is governed by the inequality:

24 $$\begin{aligned} \Vert f_\theta (x + \delta ) - f_\theta (x)\Vert&\le L \epsilon \end{aligned}$$

where *x* denotes the input, $\delta$ is any perturbation satisfying $\Vert \delta \Vert \le \epsilon$, and *L* is the Lipschitz constant of $f_\theta$. This constant essentially controls the sensitivity of the model’s output with respect to changes in the input. The robustness of the model is thus directly tied to this Lipschitz property. Because the goal of adversarial training is to optimize model robustness around an $\epsilon$-radius for any input *x*, the inequality:

25 $$\begin{aligned} \Vert f_\theta (x + \delta ) - f_\theta (x)\Vert&\le L \Vert \delta \Vert \le L \epsilon \end{aligned}$$

ensures that the model’s output does not exhibit excessive deviation under small perturbations. This result provides a robustness certificate, validating that the trained model remains stable under minor input alterations, thereby reinforcing the reliability of $f_\theta$. The overall robustness of the LGG-NRGrasp model can be further understood by examining its Lipschitz constant, which is bounded by the product of the Lipschitz constants of its principal components:

26 $$\begin{aligned} L_{\text {LGG-NRGrasp}}&\le L_{\text {GNN}} \cdot L_{\text {policy}} \cdot L_{\text {Wasserstein}}, \end{aligned}$$

where $L_{\text {GNN}}$, $L_{\text {policy}}$, and $L_{\text {Wasserstein}}$ correspond to the Lipschitz constants of the graph neural network, the policy network, and the Wasserstein distance computation, respectively. This multiplicative relationship highlights how the interplay between the model’s components contributes to its overall robustness, ensuring that perturbations in the input space are controlled in their propagation through the model. The proof follows from the composition of Lipschitz functions and the architecture of LGG-NRGrasp. We can bound the model’s overall Lipschitz constant as follows:

1. *For the graph neural network (GNN):* We use the spectral norm of the weight matrices and the properties of the activation functions to establish the Lipschitz continuity.
2. *For the policy network:* The bound is derived based on the continuity of the sigmoid activation and weight norms, providing a stable mapping from the input space to the output.
3. *For the wasserstein distance:* Utilizing the dual formulation, we impose constraints on the critic function to ensure the Lipschitz property holds across the metric space.

By integrating these component-wise constraints, we get the comprehensive Lipschitz constant for LGG-NRGrasp. The LGG-NRGrasp model’s robustness is ensured by controlling how perturbations in the input space affect the output space, constrained by the product of the Lipschitz constants of the GNN, policy network, and Wasserstein calculation. This improves the model’s performance consistency under adversarial settings.

## Experimental framework and in-depth analysis

A comprehensive experimental framework has been developed to rigorously evaluate the capabilities of the proposed LGG-NRGrasp model, with the objective of addressing the following critical research inquiries:

*Research Question 1:* How does LGG-NRGrasp’s performance in ICD code prediction stand against the benchmarked state-of-the-art automated systems, both in terms of predictive accuracy and computational efficiency?

*Research Question 2:* What algorithmic strategies can be effectively employed to enhance the generalization capacity, robustness, and holistic efficacy of the label graph generation network?

*Research Question 3:* To what degree does the NRGrasp deep graph model succeed in capturing and leveraging intricate feature hierarchies within graph data, while concurrently addressing and mitigating the persistent over-smoothing phenomenon inherent to deep graph neural network architectures?

Responding to these concerns is essential for evaluating the progress made by the LGG-NRGrasp framework, elucidating the complexities of model behavior, and improving comprehension of intricate graph-based learning paradigms.

### Implementation details

All models are implemented in PyTorch and trained on a 8 NVIDIA RTX 4090 GPUs with 24 GB memory. The hidden representation dimension is set to 128 across all layers, and dropout with a probability of 0.5 is applied to both input and intermediate layers. Xavier uniform initialization is used for all learnable weights. The models are trained for 100 epochs with early stopping patience of 10 based on validation micro-F1. We use a batch size of 64 and the Adam optimizer.

The learning rate is selected from $\{1e^{-3}, 5e^{-4}, 2e^{-4}\}$, and weight decay is chosen from $\{0, 1e^{-4}, 5e^{-4}\}$, using grid search on the validation set. The DropNode noise ratio $\delta$ is fixed at 0.2, consistent with previous studies on graph-based regularization. The consistency regularization weight $\lambda$ is tuned over $\{0.5, 1.0, 2.0\}$, and the best performance is observed at $\lambda = 1.0$. For adversarial domain adaptation, the feature alignment loss weight $\alpha$ is selected from $\{0.05, 0.1, 0.2\}$, with $\alpha = 0.1$ providing a good trade-off. The reinforcement learning discount factor $\gamma$ used in the adaptive label graph generation is fixed at 0.9.

All hyperparameters are selected using grid search on the MIMIC-III validation set and averaged across three random seeds to ensure stability. For reproducibility, we fix all random seeds and use deterministic computation wherever possible.

### Datasets and evaluation metrics

To evaluate the robustness and generalizability of the proposed LGFAT-RGCN model, we utilized two benchmark datasets, each contributing unique properties to assess different aspects of the model’s performance.

*(a) MIMIC-III*^35^: MIMIC-III, a publicly accessible clinical database, contains de-identified health data from patients admitted to intensive care units. The dataset presents notable complexity due to its extensive array of electronic health records (EHR), encompassing diverse temporal sequences of diagnoses, procedures, and lab results. This rich structure makes it an ideal testbed for evaluating graph-based learning models in medical contexts, allowing for a robust assessment of model capabilities in handling intricate and high-dimensional clinical data, and offering valuable insights into real-world applicability in healthcare settings.

*(b) FB15K-237*^36^: A subset of the Freebase knowledge base^37^, FB15K-237 represents a curated graph structure with entities and their relational edges. The dataset is derived from FB15K^21^ but is specifically modified to eliminate inverse relations between the training and test sets, thereby mitigating data leakage and providing a more rigorous benchmark for generalization capabilities. FB15K-237 spans multiple entity types and relation categories, offering a robust platform for evaluating the model’s ability to generalize across diverse knowledge graph domains.

*(c) Cora*^29^: Cora is a widely used citation network dataset, consisting of 2,708 scientific publications categorized into seven distinct classes. Each publication is represented as a 1,433-dimensional feature vector derived from a bag-of-words representation of the document. The dataset includes a citation graph where nodes represent publications and edges indicate citation relationships. The graph structure renders Cora a typical baseline for assessing graph-based algorithms, especially in node classification tasks. The intrinsic relational framework and sparsity impede the model’s capacity to acquire significant representations and generalize proficiently across graph-structured material.

### Evaluation metrics

*AUC (Area Under the Curve)*: AUC measures the area under the ROC curve, where the x-axis represents the False Positive Rate (FPR) and the y-axis represents the True Positive Rate (TPR). It is computed as $\text {AUC} = \int _{0}^{1} \text {TPR}(t) \, d\text {FPR}(t)$. A higher AUC indicates better distinction between positive and negative samples, particularly useful for imbalanced datasets.

*F1 Score (Macro & Micro)*: F1 Score is the harmonic mean of Precision and Recall, calculated as $F1 = 2 \cdot \frac{\text {Precision} \cdot \text {Recall}}{\text {Precision} + \text {Recall}}$, where $\text {Precision} = \text {TP} / (\text {TP} + \text {FP})$ and $\text {Recall} = \text {TP} / (\text {TP} + \text {FN})$. Macro F1 averages the F1 scores across all classes as $\text {Macro F1} = \frac{1}{C} \sum _{c=1}^{C} F1_c$, where *C* is the total number of classes. Micro F1 computes F1 globally across all samples as $\text {Micro F1} = 2 \cdot (\text {Micro Precision} \cdot \text {Micro Recall}) / (\text {Micro Precision} + \text {Micro Recall})$.

*P@k (Precision at k)*: P@k evaluates the proportion of relevant labels among the top *k* predictions. It is defined as $P@k = \frac{1}{|T|} \sum _{i=1}^{|T|} \frac{1}{k} \sum _{j=1}^{k} \mathbb {I}(\text {Prediction}_{i,j} \in \text {GroundTruth}_i)$, where |*T*| is the number of test samples, $\text {Prediction}_{i,j}$ is the *j*-th predicted label for the *i*-th sample, and $\mathbb {I}(\cdot )$ is an indicator function returning 1 if the prediction is correct. A higher P@k indicates better ranking performance.

*Accuracy*: Accuracy measures the proportion of correct predictions among all predictions made by the model. It is computed as $\text {Accuracy} = \text {Number of correct predictions} / \text {Total number of predictions}$. A higher accuracy indicates better alignment between the model’s predictions and the ground truth. However, it may be less informative for imbalanced datasets, requiring complementary metrics like F1 or AUC for a comprehensive evaluation.

### Comparative performance analysis (RQ1)

#### Superior performance of LGG-NRGrasp across ICD code prediction

The empirical evaluation, detailed in Table 1, highlights that LGG-NRGrasp demonstrates a significant improvement over existing baseline models, as assessed on both the MIMIC-III Full and MIMIC-III Top50 datasets. The model’s performance was rigorously evaluated using comprehensive metrics such as AUC (Area Under the Receiver Operating Characteristic Curve), F1 score, and precision at top predicted codes (P@8 for MIMIC-III Full, P@5 for MIMIC-III Top50).

In the context of the MIMIC-III Full dataset, LGG-NRGrasp delivers unparalleled performance, achieving a macro AUC of 0.958 and a micro AUC of 0.991, considerably outperforming alternative models such as BI-GRU (0.500, 0.547) and HA-GRU (0.500, 0.509). These results demonstrate that LGG-NRGrasp not only excels at discerning subtle class distinctions but also remains robust across diverse and imbalanced classes. Furthermore, the model’s macro F1 score (0.151) and micro F1 score (0.605) validate its ability to effectively balance precision and recall, particularly for underrepresented codes. This is further reflected in the P@8 metric, where LGG-NRGrasp achieves 0.864, outperforming models like LAAT (0.738) and DR-CAML (0.609), indicating enhanced prediction precision for the top ICD codes.

A similar trend is observed when evaluating the MIMIC-III Top50 subset, where LGG-NRGrasp consistently outperforms its peers. The model achieves a macro AUC of 0.931 and a micro AUC of 0.951, reflecting its superior generalization capability across frequent codes. The F1 scores, macro 0.687 and micro 0.736, provide further evidence of the model’s proficiency in optimizing the trade-off between precision and recall. Additionally, the P@5 score of 0.698 underscores LGG-NRGrasp’s robustness in predicting clinically significant codes, making it highly applicable for medical coding tasks.

**Table 1:** Performance evaluation of multi-label models on MIMIC-III Datasets.

Model	MIMIC-III Full					MIMIC-III Top50				
	AUC		F1		P@8	AUC		F1		P@5
	Macro	Micro	Macro	Micro		Macro	Micro	Macro	Micro	
BI-GRU	0.500	0.547	0.002	0.140	0.317	0.501	0.594	0.035	0.268	0.228
HA-GRU	0.500	0.509	0.017	0.004	0.296	0.500	0.436	0.072	0.124	0.205
CAML	0.895	0.986	0.088	0.539	0.709	0.875	0.909	0.532	0.614	0.609
DR-CAML	0.897	0.985	0.086	0.529	0.609	0.884	0.916	0.576	0.633	0.618
LAAT	0.919	0.988	0.099	0.575	0.738	0.925	0.946	0.666	0.715	0.675
JointLAAT	0.921	0.988	0.107	0.575	0.735	0.925	0.946	0.661	0.716	0.671
ISD	0.938	0.990	0.119	0.539	0.745	**0.935**	0.949	0.679	0.717	0.682
MSMN	0.950	**0.992**	0.103	0.584	0.752	0.928	0.947	0.683	0.725	0.697
FUSION	0.915	0.989	0.088	0.554	0.736	0.907	0.933	0.619	0.674	0.647
LGG-NRGrasp(Ours)	**0.958**	0.991	**0.151**	**0.605**	**0.864**	0.931	**0.951**	**0.687**	**0.736**	**0.698**
	± 0.001	± 0.002	± 0.001	± 0.002	± 0.001	± 0.001	± 0.001	± 0.002	±0.001	± 0.003

Significant values are in bold.

#### Enhanced handling of rare and imbalanced codes

LGG-NRGrasp demonstrates significant improvements in handling the challenges posed by rare and imbalanced ICD code distributions, which are known challenges in automatic coding tasks. As shown in Table 1, models such as CAML^38^ and DR-CAML^39^ exhibit substantial limitations in this regard, with macro F1 scores of 0.088 and 0.086, respectively, indicating their diminished capacity to predict rare codes. In contrast, LGG-NRGrasp’s robust macro metrics (0.151 for MIMIC-III Full and 0.687 for MIMIC-III Top50) indicate a stronger ability to encode sparse and low-frequency codes effectively. The model’s architecture facilitates more equitable learning across classes, optimizing for both common and rare codes without overfitting to dominant categories.

#### Comparative analysis and model constraints of GRU-based methods

The limitations of GRU-based architectures become apparent through their reported metrics in Table 1. Despite their recurrent nature, which is intended to model sequential data effectively, their performance (macro AUC of 0.500 for both BI-GRU and HA-GRU on MIMIC-III Full) falls short compared to LGG-NRGrasp (0.988). This highlights the insufficiency of traditional GRU models in capturing the complex hierarchical relationships present in ICD coding. The relatively low P@8 scores (BI-GRU^40^: 0.317, HA-GRU^41^: 0.296) further accentuate the limitations of these models in providing precise top-level code predictions. In contrast, LGG-NRGrasp achieves a P@8 score of 0.864 on MIMIC-III Full, as well as a P@5 score of 0.698 on MIMIC-III Top50, reinforcing its capability to handle these multi-faceted challenges in medical code classification.

### Generalization in sparse data contexts (RQ2)

#### Generalizability analysis

**Fig. 2:**
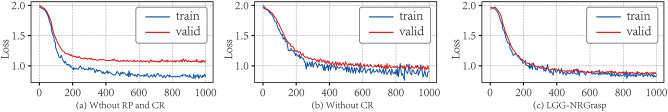
Loss comparison of LGG-NRGrasp model with and without RP and CR.

We evaluated how random propagation and consistency regularization enhance model generalizability by examining their effects on training and validation cross-entropy losses, using the FB15K-237 dataset. The gap between these losses provides insights into overfitting and generalization. Figure 2 illustrates the dynamics for LGG-NRGrasp and two key variants. When both consistency regularization (CR) and random propagation (RP) are absent, a significant divergence between validation and training losses indicates overfitting. Adding RP reduces this gap, improving generalizability. With the subsequent addition of CR, the validation loss stabilizes, closely aligning with the training loss.

#### Robustness analysis

We examine the robustness of LGG-NRGrasp by introducing perturbed graphs using two adversarial attack methodologies: the Random Attack, which perturbs the graph structure by adding random fake edges, and Metattack^42^, which strategically modifies the graph by adding or removing edges using meta-learning techniques.

**Fig. 3:**
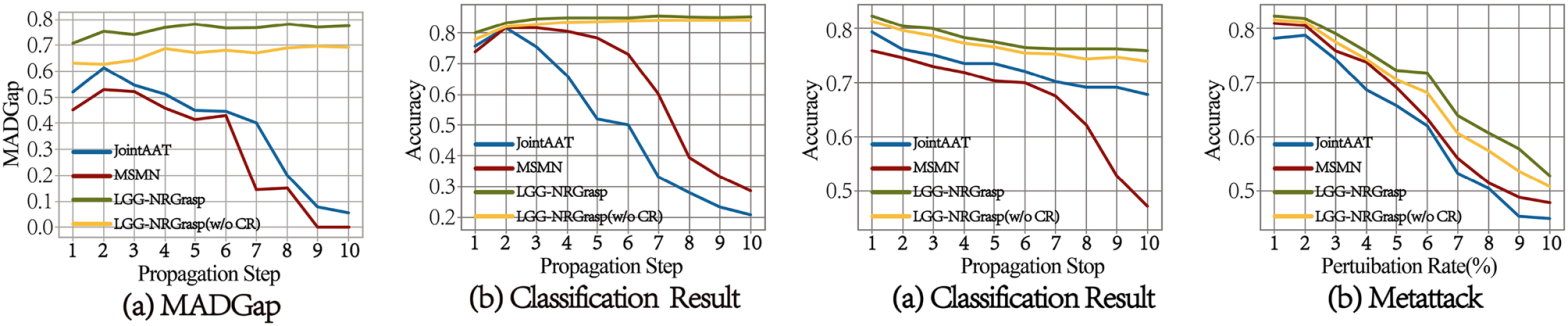
Loss comparison of LGG-NRGrasp model with and without RP and CR.

Figure 3 illustrates the classification accuracies of various models under different perturbation rates on the Cora dataset. Our analysis reveals that LGG-NRGrasp consistently outperforms both JointLAAT and MSMN across all perturbation levels for both types of attacks. Specifically, with a 10% increase in random edges, the classification accuracy for LGG-NRGrasp declines by only 7%, compared to a 12% drop for JointLAAT^4^ and a significant 37% drop for MSMN^43^. Moreover, under the Metattack scenario, the performance gap between LGG-NRGrasp and the two comparative models (JointLAAT and MSMN) widens as the perturbation rate increases. This empirical evidence underscores the robustness advantage of the LGG-NRGrasp model–whether using or omitting consistency regularization–over state-of-the-art alternatives like JointLAAT and MSMN.

#### Performance results for different configuration

We conducted an ablation investigation to meticulously analyze the distinct contributions of different components inside the LGG-NRGrasp architecture. Multiple configurations were assessed, including the elimination of consistency regularization, whereby the model depended only on supervised classification loss by assigning the regularization parameter a value of zero, so disregarding the consistency component. Another setting included implementing the DropNode(DN) operation just once every epoch, hence limiting the model’s capacity to impose low-entropy predictions for unlabeled nodes. Furthermore, we examined a configuration in which both consistency regularization(CR) and DropNode were eliminated, therefore converting the model into a streamlined version that used deterministic propagation and a multi-layer perceptron. The findings, as shown in Table 2, indicated two principal observations. Initially, all configurations with one or more components omitted shown a significant decline in performance, underscoring the critical importance of these components in guaranteeing the model’s efficacy. Secondly, even in the absence of consistency regularization, the LGG-NRGrasp model surpassed almost all non-regularization-based graph convolutional networks and the DropEdge method across three datasets. This illustrates the efficacy and importance of the suggested stochastic propagation method for improving semi-supervised graph learning.

**Table 2:** Effect of consistency regularization (CR) and DropNode (DN) on model accuracy.

Configuration	MIMIC-III	FB15K-237
Without CR	$84.4 \pm 0.5$	$73.1 \pm 0.6$
Without DN	$84.7 \pm 0.4$	$74.8 \pm 0.4$
Without CR & DN	$83.2 \pm 0.5$	$70.3 \pm 0.6$

### Advanced graphical pepresentation model experiment (RQ3)

#### Impact of adjacency matrix normalization strategies on deep graph neural networks

Adjacency matrix normalization plays a fundamental role in graph neural networks (GNNs), particularly as the depth of the network increases. Without proper normalization, deep GNNs can suffer from over-smoothing, where node embeddings become indistinguishable, leading to a deterioration in performance. To better understand how different normalization strategies affect feature propagation and model performance, we systematically evaluated four normalization approaches: *M*1,*M*2, *M*3, and *M*4, within the LGG-NRGrasp framework. These strategies were assessed across varying network depths using two benchmark datasets: FB15K-237 and Cora.

In our approach, we explore multiple normalization strategies to enhance information propagation in graph structures, ultimately proposing our adaptive method, *M*4. Starting with *M*1, which applies no normalization, we observe that treating all nodes and edges equally often results in uneven propagation, as nodes with higher degrees can disproportionately dominate the learning process. To address this, *M*2 introduces symmetric normalization by incorporating the degree matrix, balancing information flow by limiting the influence of high-degree nodes. *M*3 further refines this with row-wise normalization, ensuring that each node distributes its outgoing connections uniformly by normalizing each row of the adjacency matrix to sum to one. Our proposed method, *M*4, builds on these techniques with adaptive normalization that dynamically adjusts propagation based on both graph structure and node embeddings. This flexibility allows *M*4 to maintain stable feature propagation across deeper layers, effectively mitigating the over-smoothing challenges often seen in deeper GNN architectures, and providing a robust solution for complex graph representation tasks.

**Fig. 4:**
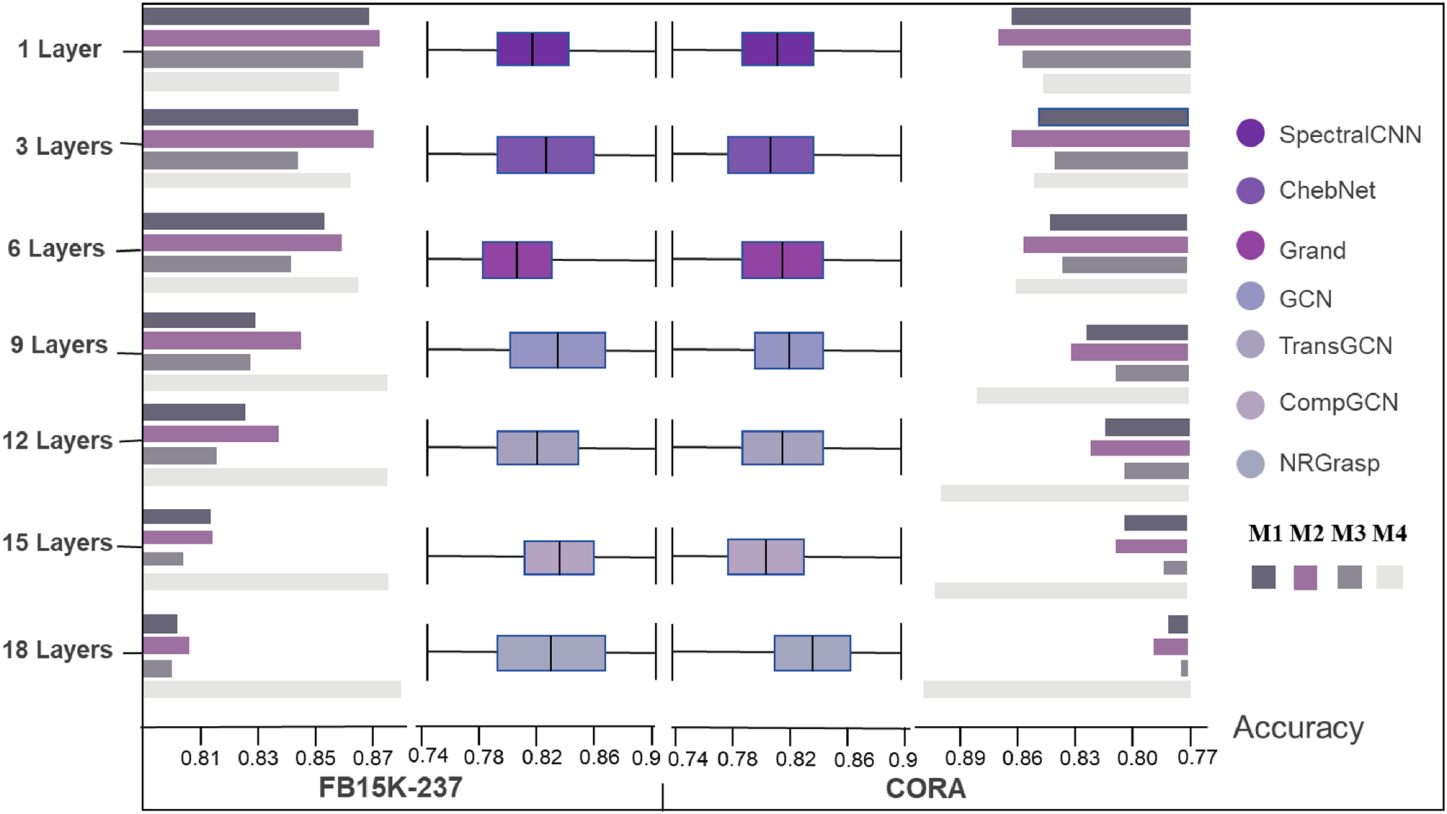
Accuracy comparison across different network depths and models on FB15K-237 and CORA Datasets.

Our experimental results, presented in Fig. 4, reveal a significant divergence in performance across these strategies as the network depth increases. At shallow depths (e.g., 1 to 3 layers), all strategies perform similarly, with accuracies ranging from 0.85 to 0.88 on the FB15K-237 dataset. However, as the network depth increases to 9 layers, *M*4 exhibits a clear advantage, achieving a peak accuracy of 0.877, compared to 0.812 for *M*1. This performance gap becomes even more pronounced at deeper layers, where the accuracies of *M*1, *M*2, and *M*3 degrade to 0.795, 0.784, and 0.848, respectively, while M4 maintains a robust accuracy of 0.883. These findings highlight the critical importance of selecting a normalization strategy that adapts effectively to deeper network architectures. *M*4’s ability to prevent the performance degradation observed in other strategies demonstrates its effectiveness in preserving feature diversity and preventing over-smoothing, making it an optimal choice for deep GNNs that require long-range dependencies to be captured accurately.

#### Comparative performance of LGG-NRGrasp across multi-relational graphs

Beyond the evaluation of adjacency matrix normalization strategies, we conducted a thorough comparative analysis of LGG-NRGrasp against state-of-the-art GNN models, including SpectralCNN, ChebNet, GCN, and GCNII. This evaluation was performed on two widely used datasets, FB15K-237 and Cora, both of which feature complex relational structures. These datasets were selected to assess the ability of each model to capture intricate multi-relational dependencies, a critical aspect of real-world graph applications, where entities are often connected through various types of relationships.

The results of our analysis, presented in Fig. 4, demonstrate that LGG-NRGrasp consistently outperforms its counterparts across a range of network depths. On the FB15K-237 dataset, LGG-NRGrasp achieves an accuracy of 0.877 at 9 layers, surpassing ChebNet, the closest competitor, which records an accuracy of 0.861. This trend is similarly observed on the Cora dataset, where LGG-NRGrasp attains a peak accuracy of 0.869, outperforming other models such as LGG-NRGrasp and CompGCN, which achieve accuracies of 0.859 and 0.854, respectively. These results underscore the ability of LGG-NRGrasp to capture multi-relational dependencies more effectively than traditional GNN models, particularly at intermediate network depths where long-range interactions must be accurately modeled.

One of the key strengths of LGG-NRGrasp is its resilience to over-smoothing, even as the network depth increases. This resilience is a crucial factor in deep GNN architectures, where models often struggle to maintain high performance due to the tendency for node embeddings to converge to similar values, thus losing their discriminative power. LGG-NRGrasp addresses this challenge through the integration of advanced adjacency matrix normalization and adaptive learning mechanisms, enabling it to preserve meaningful feature representations across multiple layers. The robust performance of LGG-NRGrasp across both FB15K-237 and Cora datasets highlights its potential as a powerful tool for handling complex graph-based tasks in multi-relational settings. The ability to maintain high accuracy in these challenging environments positions LGG-NRGrasp as a leading model for applications that require the modeling of intricate topologies and diverse relational structures.

### Computational complexity and efficiency analysis

Compared to traditional graph neural network architectures such as GCN and GAT, LGG-NRGrasp introduces additional computational overhead due to two key components: the reinforcement-based label graph generation and the mixed-order feature propagation with stochastic node embedding.

The label graph generator uses a trajectory-based policy gradient method, specifically REINFORCE, with per-episode complexity of $\mathcal {O}(T \cdot |E|)$, where *T* represents the trajectory length and |*E*| is the number of candidate label edges. Given that |*E*| scales quadratically with the number of labels in dense taxonomies, this step incurs a non-trivial computational cost. However, the use of edge sampling techniques and early stopping criteria significantly reduces redundant exploration, ensuring that the overall complexity remains sublinear with respect to full edge enumeration.

The DropNode-based stochastic embedding module performs node-wise masking followed by feature propagation through a mixed adjacency matrix $\bar{A} = \sum _{k=0}^K \frac{1}{K+1} \hat{A}^k$, which involves *K* sparse matrix multiplications. The overall complexity of this stage is $\mathcal {O}(K \cdot |\mathcal {E}| + K \cdot d \cdot |\mathcal {V}|)$, where $|\mathcal {E}|$ is the number of edges, $|\mathcal {V}|$ is the number of nodes, and *d* is the feature dimensionality. This complexity is comparable to methods like MixHop^10^, but more efficient than DropEdge^7^ as in Table 3, which perturbs edge-level structure during each batch and can disrupt sparsity patterns during training.

LGG-NRGrasp incurs a training time that is approximately 1.4$\times$ greater than that of GCN and 1.2$\times$ greater than DropEdge on the MIMIC-III dataset. However, it demonstrates significantly higher robustness in the presence of domain shifts. Inference time remains comparable to GAT, as label graph construction is executed offline before deployment. Consequently, the efficiency-robustness trade-off makes LGG-NRGrasp particularly well-suited for clinical environments where distribution shifts and label noise are common.

**Table 3:** Computational complexity versus performance comparison of graph neural network methods.

Method	Computational complexity	Training time	Performance (micro-F1)
GCN	$\mathcal {O}(d \cdot |\mathcal {E}|)$	Fast	Baseline
DropEdge	$\mathcal {O}(d \cdot |\mathcal {E}|) + \text {edge masking overhead}$	Moderate	Improved with domain shift
GAT	$\mathcal {O}(d^2 \cdot |\mathcal {E}|)$	Slow (Attention overhead)	Moderate
LGG-NRGrasp	$\mathcal {O}(T \cdot |E|) + \mathcal {O}(K \cdot |\mathcal {E}|)$	Slow, but robust	textbfSuperior in domain shift

## Limitations and future directions

While LGG-NRGrasp demonstrates robustness and strong generalization across heterogeneous clinical graph datasets, certain limitations are inherent to its design assumptions. The framework presupposes an implicit structural coherence between the evolving label graph and the predictive task, an assumption that may not hold in low-resource or institutionally fragmented coding systems. In such cases, the reinforcement-based label graph generation may suffer from representational collapse, particularly when label dependencies are weakly observed or inconsistently encoded. Moreover, while trajectory-level optimization supports flexible policy learning, it introduces high-variance credit assignment and non-trivial computational overhead, which can constrain scalability in deployment-critical settings.

The model also operates under the assumption that domain shifts manifest primarily as smooth distributional drifts in the feature space. However, clinical distribution shifts often exhibit semantic discontinuities–such as policy-driven label redefinitions or emergent comorbidity patterns–that may not be adequately captured by transport-based adversarial adaptation alone. The Wasserstein distance, while geometry-aware, does not explicitly account for label space misalignment or evolving taxonomies, both of which are prevalent in real-world healthcare systems, particularly under federated or cross-institutional settings.

Another limitation stems from the model’s uni-modal foundation. Current inputs rely exclusively on textual abstractions from clinical notes, thereby overlooking multimodal signals increasingly available in modern EHRs, such as temporal dynamics, imaging, laboratory results, and structured ontological metadata. This restricts the semantic richness and causal grounding of the learned representations. Although theoretical convergence guarantees are established, real-world robustness under adversarial or policy-induced distributional shifts remains empirically underexplored.

Future extensions of LGG-NRGrasp will focus on integrating symbolic priors and structure-aware constraints derived from clinical ontologies (e.g., SNOMED CT, ICD-11) into the graph generation process, enabling greater semantic alignment and interpretability. Hybridizing the model with foundation-level clinical LLMs could enhance its capacity for zero-shot generalization across unseen diagnostic codes. Furthermore, enabling dynamic graph evolution, uncertainty-aware policy refinement, and causal intervention modeling may facilitate continuous learning under real-time data streams, contributing toward the development of robust, adaptive, and explainable clinical AI systems.

Additionally, although LGG-NRGrasp demonstrates strong predictive performance, its current design does not explicitly address real-time applicability within clinical information systems. Inference latency, computational efficiency, and hardware feasibility are critical in practical deployment scenarios, especially where ICD coding must be performed under time constraints. These aspects fall outside the scope of the present study, which focuses on algorithmic innovation and robustness. Future work will investigate the integration of LGG-NRGrasp with hardware-accelerated platforms tailored for GNN inference. Notably, recent developments such as HyGCN^44^, AWB-GCN^45^, GraphACT^46^, and GCNAX^47^ offer specialized architectures for efficient and scalable graph computation. GCNAX, in particular, introduces a configurable systolic array design that optimizes sparse matrix operations and supports real-time graph workloads. By leveraging such accelerators, future implementations of LGG-NRGrasp may be adapted for low-latency, high-throughput environments, paving the way for deployment in edge computing or in-memory computing scenarios within hospital information systems^48,49^.

## Conclusion

In this study, we present LGG-NRGrasp, a robust framework for International Classification of Diseases (ICD) classification, addressing challenges within sparse and hierarchical data environments. Our experiments confirm the model’s superior performance across metrics such as AUC and micro-F1, outperforming state-of-the-art approaches while enhancing robustness and generalization. The ablation study highlights the critical roles of consistency regularization and DropNode augmentation in model efficacy. LGG-NRGrasp’s adaptability to complex graph structures and over-smoothing mitigation demonstrates its potential for broader applications in hierarchical classification tasks beyond healthcare. Future directions include exploring multi-modal data integration and extending its capabilities to real-time clinical decision systems.

## Data Availability

This is our code file: https://anonymous.4open.science/r/SR-3F00/. These are our dataset files: https://physionet.org/content/mimiciii/1.4/. https://paperswithcode.com/dataset/fb15k-237. https://paperswithcode.com/dataset/cora.
